# Evaluation of intraocular gas using magnetic resonance imaging after pars plana vitrectomy with gas tamponade for rhegmatogenous retinal detachment

**DOI:** 10.1038/s41598-020-58508-3

**Published:** 2020-01-30

**Authors:** Makoto Gozawa, Masayuki Kanamoto, Shota Ishida, Yoshihiro Takamura, Kentaro Iwasaki, Hirohiko Kimura, Masaru Inatani

**Affiliations:** 1 Department of Ophthalmology, Faculty of Medical Sciences, University of Fukui, 23-3 Shimoaizuki, Matsuoka, Eiheiji, Yoshida, Fukui, 910-1193 Japan; 2 Radiological Center, University of Fukui Hospital, 23-3 Shimoaizuki, Matsuoka, Eiheiji, Yoshida, Fukui, 910-1193 Japan; 3 Department of Radiology, Faculty of Medical Sciences, University of Fukui, 23-3 Shimoaizuki, Matsuoka, Eiheiji, Yoshida, Fukui, 910-1193 Japan

**Keywords:** Magnetic resonance imaging, Retinal diseases

## Abstract

We used magnetic resonance imaging (MRI) to assess how a patient’s posture affects intraocular gas changes and whether the postoperative prone position is required after pars plana vitrectomy (PPV) with gas tamponade for rhegmatogenous retinal detachments (RRDs). Eight patients with RRDs who underwent PPV combined with cataract surgery with gas tamponade were prospectively included. They underwent MRI examination both in the prone and supine positions. We separated the retina into four parts: superior–posterior, superior–anterior, inferior–posterior, and inferior–anterior. We then calculated the gas contact rate as (the length of the retina contacting the gas in each retinal part) divided by (the length of each retinal part) × 100% in both the prone and supine positions. The mean gas contact rate of the superior–anterior part of the retina was significantly higher (P = 0.006) in the supine position than in the prone position. The mean gas contact rate of the inferior–anterior part of the retina was also significantly higher (P = 0.0004) in the supine position than in the prone position. We believe that if all retinal breaks were located anterior to the equator, the supine position may provide better tamponade gas coverage for the breaks than the prone position. Although potential postoperative complications caused by the supine position require careful attention, our result may shorten the duration of postoperative prone position and may decrease the patients’ discomfort after PPV with gas tamponade for RRDs.

## Introduction

Pars plana vitrectomy (PPV) with gas tamponade has recently become popular as the first-line therapy for rhegmatogenous retinal detachments (RRDs)^1–3^. The rationale for using long-acting gas for the management of retinal breaks with PPV is that intraocular gas bubbles act as a tamponade that prevent intravitreal fluid from entering the retinal break and accumulating in the subretinal space. Successfully repairing RRDs using long-acting gas relies on its ability to close retinal breaks by occlusion^4,5^. Therefore, it is important to maintain an appropriate postoperative posture allowing the tamponade gas to make contact with retinal breaks.

The maintenance of the postoperative position after PPV with gas tamponade, particularly for inferior breaks, remains controversial. At present, the prone position is recommended^6–11^ for preventing postoperative complications, such as retinal translocation, glaucoma, pupillary block, anterior chamber shallowing, IOL dislocation/iris capture, and IOL/cornea touch; however, medical, physical, or mental conditions prevent patients from maintaining the prone position after surgery^12–14^. In this regard, some recent studies have demonstrated that a strict postoperative prone position is not needed for PPV with gas tamponade for RRDs. These studies included a consecutive noncomparative study of pseudophakic eyes^15^, a comparative study of PPV using long-acting gas^16^, and a retrospective comparative study of PPV with cataract surgery for RRDs^17^. However, these results do not fully explain why the postoperative prone position is not required. Shiraki *et al*. discussed that intraocular gas might not come into contact with the breaks during incomplete prone positioning. In addition, Bell’s phenomenon might result in the exposure of breaks to intravitreal fluid and not to gas, especially when the eyes are shut^17^. Tetsumoto *et al*. reported that the gas may act better on the peripheral retina in the supine position than in the prone position^18^. However, standard ophthalmic examinations, such as slit lamp examination, anterior segment optical coherence tomography or ultrasound biomicroscopy, do not reveal whether the tamponade gas closes the breaks when patients are in prone position or when the eyes are shut.

Magnetic resonance imaging (MRI) is a useful and noninvasive tool for the imaging of intraocular lens, scleral buckles glaucoma filtration devices, bleb fluid structure, and intraocular tamponade agents^19–22^. However, there are no reports evaluating the position of intraocular tamponade gas using MRI after PPV with gas tamponade for RRDs. Therefore, this study aimed to use MRI to assess how a patient’s posture affects intraocular gas changes and whether the postoperative prone position is required after PPV with gas tamponade for RRDs.

## Methods

### Patient selection

This study was approved by the Institutional Review Board of the University of Fukui Hospital, Fukui, Japan, and adhered to the tenets of the Declaration of Helsinki. The protocol and the possible risks and benefits of the interventions were explained to all participants before enrollment. Written informed consent was obtained from all participants. This study was registered with the University Hospital Medical Information Network-Clinical Trials Registry of Japan (ID UMIN 000032491; date of access and registration, May 7, 2018). Patients with RRDs with phakic eyes who underwent PPV combined with cataract surgery using long-acting SF6 (20%) gas tamponade were recruited from University of Fukui Hospital between June 25, 2018 and June 10, 2019. Patients with contraindications for MRI with intolerance, cardiac pacemakers, or presence of tattoos or metal in their bodies or those who had strabismus or showed postoperative complications, such as a retinal redetachment vitreous hemorrhage at the time of taking MRI images, were excluded.

### Surgical procedures

One surgeon (MG) performed all surgeries, which were performed as previously described^17^. In brief, four-port PPV was performed using chandelier and a wide-angle viewing system (Resight, Carl Zeiss Meditec, Germany) with a 25-gauge system (Constellation, Alcon Laboratories Inc. Fort Worth, TX, USA). Before vitrectomy, cataract surgery (phacoemulsification and intraocular lens [IOL] implantation) with 2.4 mm bent transconjunctival single-plane sclerocorneal incision was performed using the same machine for all phakic eyes. The size of the circular continuous capsulorhexis was adjusted to completely cover the three-piece IOL (X-70, Santen, Osaka, Japan), which was implanted. After core vitrectomy, triamcinolone acetonide (MaQuaid, Wakamoto Pharmaceutical, Tokyo, Japan) was sprayed toward the optic disc and the posterior retinal surface to ascertain the presence of a posterior vitreous detachment. The peripheral vitreous was shaved at 360° as much as possible under scleral indentation. In some eyes, liquid perfluorocarbon (Perfluoron, Alcon Laboratories Inc.) was used to stabilize the detached retina. After vitreous shaving, fluid-air exchange and endophotocoagulation were performed around all retinal breaks and lattice degeneration. After the retina was reattached completely, an air-gas (20% sulfur hexafluoride [SF6]) exchange was performed. Any sclerotomy sites that were found to leak at the end of the surgery were sutured with 8-0 vicryl suture. All patients were instructed to maintain the prone position immediately after surgery and on the day of the surgery, followed by the appropriate position based on the location of the breaks. For example, patients with inferior breaks were instructed to maintain the supine position, whereas those without inferior breaks were instructed to maintain the supine or lateral position.

### Magnetic resonance imaging

**Figure 1:**
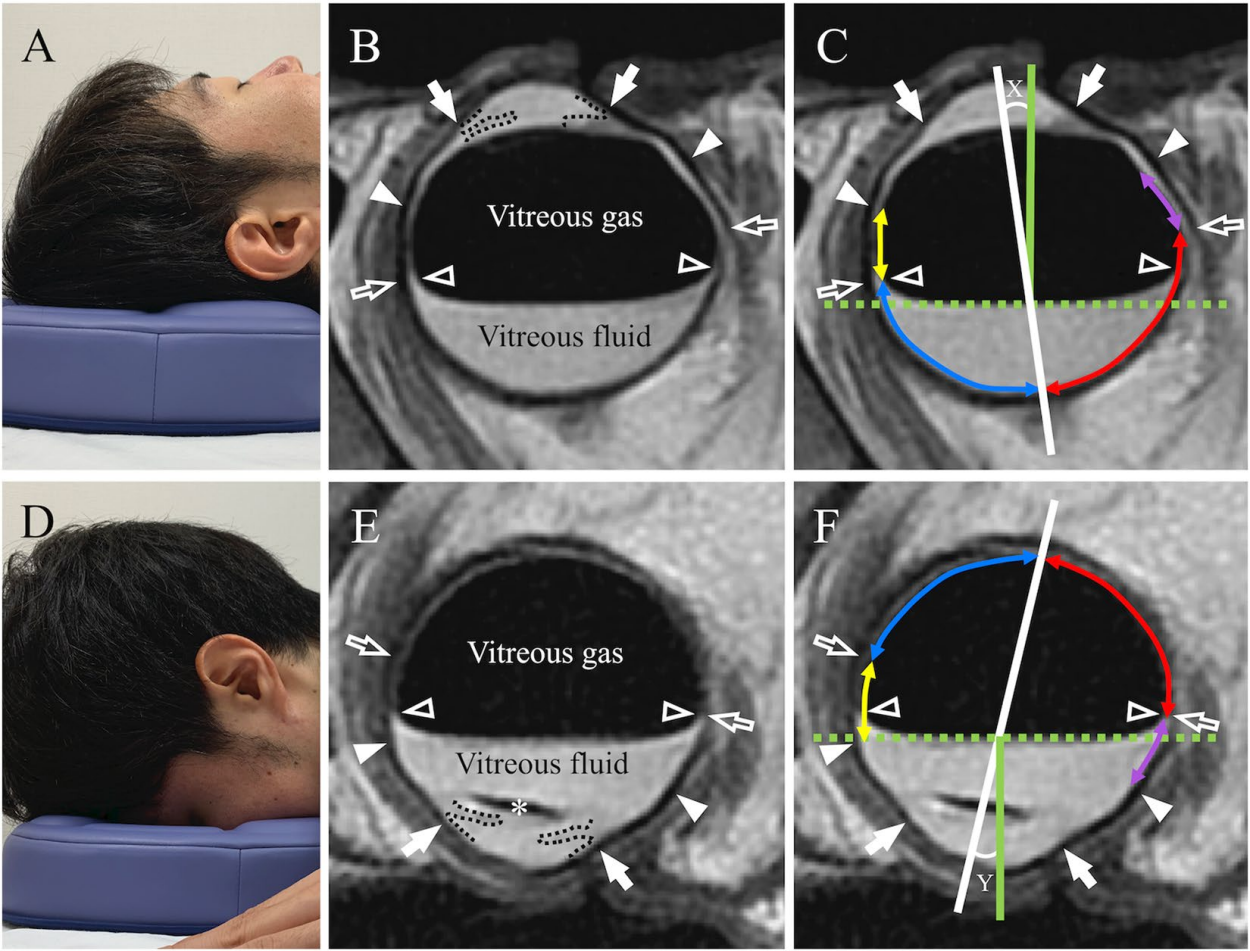
Positions during MRI examination and MRI images in the supine and prone positions. The supine (**A**) and prone (**D**) positions during MRI; (**B,C,E,F**) MRI images of case 5; (**B,C**) in supine position and (**E,F**) in prone position. White arrows, white arrow heads, and white open arrow indicate corneal limbus, ora serrata (7.0 mm posterior to the limbus) and equator line (13.5 mm posterior to the limbus), respectively. White open arrow head indicates the ends of the retina contacting with the gas. Black dotteds line indicate the margin of angle and iris. IOL could be observed (*). White solid line, green dot line, and green solid line indicate pupillary axis, horizontal line, and perpendicular line to the ground, respectively. X and Y are the supraduction angle to the perpendicular line in supine and prone position, respectively. The anterior part to the equator is shown as purple and yellow line, which indicates the inferior–anterior and superior–anterior parts of the retina, respectively. The posterior part to the equator is shown as red and blue line. Red and blue lines indicate the inferior–posterior and superior–posterior parts of the retina, respectively.

All patients underwent MRI examination both in the prone and supine positions. All scans were obtained at four days after surgery. The postures during MRI are shown in Fig. 1. Patients used EXGEL face mat and bust mat (KAJI CORPORATION, Shimane, Japan) as they do not have metallic parts. Images were obtained using a 1.5 Tesla scanner (Optima 450w; GE Healthcare, Milwaukee, WI) in combination with a 16-element phased-array flex coil with fast imaging sequences employing periodically rotated overlap ping parallel lines with enhanced reconstruction (PROPELLER). The imaging parameters were as follows: repetition time, 3000 ms; echo time, 90 ms; field-of-view, 140 mm; matrix size, 320 × 320; and a slice thickness of 3.0 mm with intersection gap of 0.5 mm, echo train length, 29; bandwidth, 50 Hz/pixel, number of excitations, 4. The specific absorption rate was calculated to be <2.0 W/kg, which is classified as “less invasive” for patients.

### Measurement of gas volume to the vitreous cavity

MRI data were saved as DICOM files and analyzed using the ImageJ software (available at http://rsb.info.nih.gov/ij/; developed by Wayne Rasband, MD, PhD, National Institutes of Health, Bethesda, MD). A threshold was applied to identify individual vitreous cavity and intraocular gas in each slice, and the areas of vitreous cavity and intraocular gas was calculated using the ImageJ software. For an automatic threshold selection, Otsu thresholding^23^ was chosen. The mean values of vitreous cavity and intraocular gas were integrated and the gas volume (%) to the vitreous cavity was calculated.

### Measurement of gas contact rates of the retina

Of all patient scanned images, we chose the slice in which the axial length was the same as that acquired from IOLMaster (Carl Zeiss Meditec, Germany) before surgery. First, we defined the intersection of the perpendicular line from the angle with the ocular surface as the surgical limbus^24^. Next, we defined the position of ora serrata and the equator defined by vortex vein ampullae as 7 mm and 13.5 mm posterior to the limbus respectively as previously reported^25^. Then, we separated the retina into superior and inferior parts and further, we separated the superior and inferior part to anterior (from the equator to the ora serrata) and posterior (from the intersection of pupillary axis and the retina to the equator) parts, respectively. This was done because retinal tears are usually found around the equator or in the peripheral retina defined as the area between the vortex vein ampullae and the ora serrata^26^. Therefore, the superior and inferior retina were separated into four parts: superior–posterior, superior–anterior, inferior–posterior, and inferior–anterior. The superior–posterior and superior–anterior parts of the retina are shown as blue and yellow lines, respectively, whereas the inferior–posterior and inferior–anterior parts are shown as red and purple lines, respectively, in Fig. 1. We then measured the length of each retinal part and the length of the retina contacting the gas in each retinal part by manual tracing using the ImageJ software. We calculated the gas contact rates as (the length of the retina contacting the gas in each retinal part) divided by (the length of each retinal part) × 100% both in the prone and supine positions.

### Statistical analyses

Statistical analyses were performed using JMP 14 (SAS institute Inc., Tokyo, Japan). The Wilcoxon signed-rank test was performed to compare the preoperative best corrected visual acuity (BCVA) (mean ± SE) to postoperative BCVA and the Mann-Whitney *U* nonparametric test was performed to compare the gas volume, angle of supraduction to the perpendicular line, and gas contact rates with retina between prone and supine position. P values < 0.05 were considered statistically significant.

## Results

### Patient characteristics

Eight phakic eyes of eight patients (four men and four women) with RRDs were included in this prospective study. No patient was excluded from this study. Table 1 summarizes the patient characteristics. Mean patient age was 59.8 years (range, 52–66). Mean axial length was 25.4 mm (range, 23.8–26.7). The mean number of quadrants affected and retinal breaks were 1.9 (range, 1–4) and 2.8 (range, 1–8), respectively. All breaks of all eyes were anterior to the equator — three eyes had breaks in the inferior quadrant, and five eyes had multiple breaks; two cases had the macula detached preoperatively.

**Table 1:** Patient characteristics.

Case	Age, Years	Sex	Eye	Lens Status	AL (mm)	Quadrant	Number of breaks	Break Postion in Relation to Equator	Break Position (o’-clock)	Macula
1	63	M	R	Phakic	23.8	1	3	Anterior	5, 5:30, 6	On
2	54	F	L	Phakic	26.4	2	1	Anterior	11	Off
3	61	F	R	Phakic	25.7	2	2	Anterior	9, 12	On
4	66	M	L	Phakic	25.9	4	8	Anterior	2, 2:30, 5, 5:30, 6, 7, 11, 12	On
5	57	F	L	Phakic	24.5	2	3	Anterior	2, 11:30, 12	Off
6	65	M	R	Phakic	25.8	1	1	Anterior	11	On
7	60	F	R	Phakic	24.1	1	1	Anterior	2:30	On
8	52	M	R	Phakic	26.7	2	3	Anterior	5:30, 6, 10	On
Mean ± SE	59.8 ± 1.8				25.4 ± 0.4	1.9 ± 0.4	2.8 ± 0.8			

AL = Axial length.

### Postoperative outcomes

Table 2 shows the postoperative outcomes. Initial reattachment was achieved in all cases. The mean final postoperative BCVA (0.11 ± 0.07) improved significantly from the mean preoperative BCVA (0.82 ± 0.27) (p = 0.012). Intraocular pressure elevations over 22 mmHg after surgery were observed in two eyes, and IOP under 21 mmHg was maintained with anti-glaucoma eye drops, which could then be withdrawn. IOL capture and proliferative vitreoretinopathy (PVR) did not occur in any eyes.

**Table 2:** Postoperative outcome.

Case	Tamponade agent	The day of taking MRI image after surgery	BCVA (logMAR)		Initial reattachment	IOL optic capture after surgery	PVR after surgery
			Preoperative	Postoperative			
1	SF6 (20%)	4	0.10	−0.08	Yes	No	No
2	SF6 (20%)	4	1.22	0.52	Yes	No	No
3	SF6 (20%)	4	0.40	0.00	Yes	No	No
4	SF6 (20%)	4	0.40	0.05	Yes	No	No
5	SF6 (20%)	4	0.82	0.22	Yes	No	No
6	SF6 (20%)	4	2.00	0.05	Yes	No	No
7	SF6 (20%)	4	−0.08	0.10	Yes	No	No
8	SF6 (20%)	4	1.70	0.00	Yes	No	No
Mean ± SE			0.82 ± 0.27	0.11 ± 0.07			

SF6 = Sulphur hexafluoride; BCVA = Best Corrected Visual Acuity; IOL = Intraocular lens; PVR = Proliferative vitreoretinopathy.

### MRI measurement

#### Gas volume

The mean gas volume (%) to the vitreous cavity in prone and supine positions was 60.1% (range, 34.9–70.8) and 60.0% (range, 34.5–70.2), respectively (Table 3, Supplementary files 1 and 2). There was no significant difference between the two positions (P = 1.0).

**Table 3:** Gas volume and the supraduction angle in each case.

Case	Gas volume (% to vitreous cavity)		Angle (°) to the perpendicular line	
	Prone position	Supine position	Prone position	Supine position
1	58.4	58.8	23.6	−16.1
2	59.7	59.0	20.7	13.2
3	58.2	58.3	29.9	6.2
4	70.8	70.2	12.7	−6.3
5	67.6	67.3	14.5	9.7
6	34.9	34.5	9.5	9.5
7	62.4	62.6	11.7	−2.2
8	68.7	69.0	6.3	0
Mean ± SE	60.1 ± 4.0	60.0 ± 4.0	16.1 ± 2.8	1.8 ± 3.5

SE = standard error.

#### Supraduction angle

The supraduction angle (°) to the perpendicular line showed that in the prone position, all eyes were in supraduction. In supine position, three eyes (case 1, 4, and 7) were in infraduction and the others were in supraduction. The mean value of the supraduction angle to the perpendicular line was significantly larger (P = 0.012) in prone position (16.1° range, 6.3–29.9) than in supine position (1.8° range −16.1–13.2) (Table 3 and 4).

**Table 4:** The result of the comparison between prone and supine position.

	Prone Position	Supine Position	P Value
Gas volume (% to vitreous cavity)	60.1 ± 4.0	60.0 ± 4.0	1.0
Angle of Supraduction to the perpendicular line(°)	16.1 ± 2.8	1.8 ± 3.5	0.012
Gas contact rates with retina (%)			
Superior-posterior	98.2 ± 1.8	5.2 ± 3.2	0.0004
Superior-anterior	40.3 ± 10.1	89.1 ± 10.7	0.006
Inferior-posterior	84.9 ± 5.5	7.9 ± 3.1	0.0008
Inferior-anterior	1.1 ± 1.1	90.1 ± 5.5	0.0004

Values are mean ± standard error.

#### Gas contact rates with retina

Supplementary files 1 and 2 show the gas contact rates of the retina in each case in the prone supine positions, respectively. Table 4 shows the results of statistical analysis of the comparison of the gas contact rates of retina between the supine and prone positions. The mean gas contact rate of the superior–anterior part of the retina was significantly higher (P = 0.006) in the supine position (89.1% range, 13.9–100) than in the prone position (40.3% range, 0–73.6). Furthermore, the mean gas contact rates of the superior–posterior part was significantly higher (P = 0.0004) in the prone position (98.2% range, 86.0–100) than in the supine position (5.2% range, 0–24.2). The mean gas contact rate of the inferior–anterior part of the retina was significantly higher (P = 0.0004) in the supine position 90.1% (range, 62.0–100) than in the prone position (1.1% range, 0–8.4). The mean gas contact rate of the inferior–posterior part of the retina was significantly higher (P = 0.0008) in the prone position (84.9% range, 65.8–100) than in the supine position 7.9% (range, 0–18.7).

## Discussion

To the best of our knowledge, this is the first study to observe intraocular gas using MRI both in the supine and prone positions. We could clearly determine the changes in the intraocular gas as well as the location of the gas in the vitreous space in both positions using the PROPELLER mode, even if the patients closed their eyes. In this study, we demonstrated that the mean gas contact rates of the superior–anterior and inferior–anterior parts of the retina were significantly higher in the supine position than in the prone position. On the other hand, the mean gas contact rates of the superior–posterior and inferior–posterior parts of the retina were significantly higher in the prone position than in the supine position. Our study is the first study to measure the supraduction angle of the eyeball to the perpendicular line both in the supine and prone positions using MRI. We demonstrated that the supraduction angle while closing eyes was positive even if the patients superficially maintained a strict prone position.

The rationale for using long-acting tamponade gas in the management of retinal breaks with PPV was based on the theory that the intraocular gas bubble provides tamponade to the retinal breaks so that intravitreal fluid cannot enter the break, and therefore, accumulates in the subretinal space^27,28^. Yoon YH *et al*. reported that after laser photocoagulation of the retina, the adhesive force was transiently reduced but increased beyond normal and remained twice the normal between 3 days and 4 weeks^29^, indicating that all retinal breaks should be closed with the intraocular gas after PPV with gas tamponade until the strength of the adhesion by photocoagulation is sufficient to prevent retinal redetachment.

It is well known that retinal tears are usually found around the equator or in the peripheral retina defined as the area between the vortex vein ampullae and the ora serrata^26^. The current study demonstrated that the mean gas contact rates of the superior–anterior and inferior–anterior parts of the retina were significantly higher in the supine position than in the prone position. Therefore, the supine position was the better than the prone or lateral position to close all breaks in the anterior part, when the retinal breaks were located only in inferior, in bilateral, in both superior and inferior, and in all quadrants. When the breaks were limited in the anterior part of each quadrant except inferior retina, the position which avoided the original retinal breaks were in the lowest position was the best position, but supine position was also sufficient to close all the breaks.

On the other hand, supine positioning itself potentially may be associated with postoperative complications, such as pupillary block, anterior chamber shallowing, IOL dislocation/iris capture, and IOL/cornea touch. Shiragami *et al*. reported the importance of a strict prone position after PPV with tamponade gas to prevent postoperative complications in eyes with RRDs and demonstrated that the retina may move downward after surgery and that unintentional postoperative retinal translocation may easily occur if the extent of the retinal detachment was large or if macular detachment was present, requiring a strict prone position to prevent retinal translocation^6^. Otsuka K *et al*. reported that there was no difference in postoperative complications between patients in a strict postoperative prone position in comparison to patients in a prone position on the day of the surgery followed by supine positioning^30^. Therefore, considering these reports and the results of the current study, if all the retinal breaks were located anterior to the equator, then patients should keep a strict prone positioning immediately after and on the day of the surgery, followed by supine positioning to prevent postoperative complications.

On the other hand, our current study showed that the mean gas contact rates of the superior–posterior and inferior–posterior parts of the retina were significantly higher in the prone position than in the supine position. Therefore, when all of the retinal breaks are limited to the posterior part of the retina, the prone position was better than the supine position. In addition, when the breaks were located both in the posterior and anterior parts of the retina of the inferior or bilateral quadrant, buckles or silicon oil may be needed as a tamponade agent. However, when the breaks were located both in the posterior and anterior parts of the unilateral or superior quadrant, the position avoiding the original retinal breaks in the lowest position was sufficient to close the breaks.

In both supine and prone positions, the mean value of the supraduction angle to the perpendicular line was positive. This was probably due to Bell’s phenomenon because patients were instructed to keep their eyes closed during MRI examination. In addition, the supraduction angle was significantly larger in the prone position than in the supine position, which was probably because in the prone position, the patients’ forehead was pushed back by the face mat and the head was tilted back. On the other hand, in the supine position, the back side of the patient’s head was pushed back by the face mat and the head was tilted forward. However, in spite of the difference in supraduction angle between prone and supine position, the mean gas contact rates with the anterior part of the retina was significantly higher in supine than in the prone position.

In cases 1, 4, and 7, the supraduction angle to the perpendicular line was negative. Despite this, the gas contact rates with the anterior part of the retina were markedly higher in supine than in the prone position. This indicated that if the patients were looking slightly downward, supine position was better than prone position to cover all the breaks located anterior to the equator. In case 6, the gas contact rates with the anterior part of the retina were markedly lower than other cases, especially superior retina, which was because the gas volume was much smaller than the other cases. This indicated that small gas volume was not sufficient to close the breaks, regardless of position.

This study has some limitations. First, in consideration of patient’s discomfort, MRI was not performed immediately after surgery. Therefore, the gas contact rates with retina in the immediate postoperative period remain unclear. The sample size of this study was relatively small. Further, eyes with axial length >27.0 and <22.0 mm or those with special shapes, such as staphyloma, were not included. In addition, we did not consider that the ora is at different locations based on the meridian when we determined the location of the ora on the MRI images. In practice, many post-vitrectomy patients are instructed to maintain the side-lying position rather than the prone or supine position; however, this was not evaluated in this study. Supine positioning itself may be associated with potential complications, such as postoperative retinal translocation, glaucoma, pupillary block, anterior chamber shallowing, IOL dislocation/iris capture, and IOL/cornea touch. Additional examinations are required to assess the safety of the supine position after PPV with gas tamponade for RRD.

## Conclusion

In conclusion, our study is the first to evaluate the position of the intraocular gas and gas contact rates of the retina in the prone and supine positions using MRI after PPV with gas tamponade for RRDs. We demonstrated that if all retinal breaks were located anterior to the equator, the supine position may provide better coverage for the breaks by tamponade gas than the prone position.

## Supplementary Information

Supplementary Information 1.

Supplementary Information 2.
