# Supplementary material for: Evaluation of intraocular gas using magnetic resonance imaging after pars plana vitrectomy with gas tamponade for rhegmatogenous retinal detachment
Source: Sci Rep. 2020 Jan 30;10:1521. doi: 10.1038/s41598-020-58508-3 (PMC6992615; doi:10.1038/s41598-020-58508-3)
Supplement: Supplementary file 1 — Supplementary Information 1. [file 41598_2020_58508_MOESM1_ESM.docx]

Evaluation of intraocular gas using magnetic resonance imaging after pars plana vitrectomy with gas tamponade for rhegmatogenous retinal detachment

Makoto Gozawa M.D.^1^, Masayuki Kanamoto Ph.D.^2^, Shota Ishida Ph.D.^2^, Yoshihiro Takamura M.D., Ph.D.^1^, Kentaro Iwasaki M.D.^1^, Hirohiko Kimura M.D., Ph.D.^3^, Masaru Inatani M.D., Ph.D^1^

^1^ Department of Ophthalmology, Faculty of Medical Sciences, University of Fukui, 23-3 Shimoaizuki, Matsuoka, Eiheiji, Yoshida, Fukui 910-1193, Japan

^2^ Radiological Center, University of Fukui Hospital, 23-3 Shimoaizuki, Matsuoka, Eiheiji, Yoshida, Fukui 910-1193, Japan

^3^ Department of Radiology, Faculty of Medical Sciences, University of Fukui, 23-3 Shimoaizuki, Matsuoka, Eiheiji, Yoshida, Fukui 910-1193, Japan

**Supplementary file 1 Gas contact rates in each case in prone position**

|  | Prone Position | | | | |
| --- | --- | --- | --- | --- | --- |
| Case | Superior-posterior | Superior-anterior |  | Inferior-posterior | Inferior-anterior |
| 1 | 100 | 73.6 |  | 65.8 | 0 |
| 2 | 100 | 13.3 |  | 77.7 | 0 |
| 3 | 100 | 71.5 |  | 73.2 | 0 |
| 4 | 100 | 33.7 |  | 100 | 8.4 |
| 5 | 100 | 69.0 |  | 100 | 0 |
| 6 | 86.0 | 0 |  | 66.4 | 0 |
| 7 | 100 | 21.3 |  | 96.2 | 0 |
| 8 | 100 | 40.1 |  | 100 | 0 |
| Mean ± SE | 98.2 ± 1.8 | 40.3 ± 10.1 |  | 84.9 ± 5.5 | 1.1 ± 1.1 |

SE = standard error
