# Supplementary material for: Evaluation of intraocular gas using magnetic resonance imaging after pars plana vitrectomy with gas tamponade for rhegmatogenous retinal detachment
Source: Sci Rep. 2020 Jan 30;10:1521. doi: 10.1038/s41598-020-58508-3 (PMC6992615; doi:10.1038/s41598-020-58508-3)
Supplement: Supplementary file 2 — Supplementary Information 2. [file 41598_2020_58508_MOESM2_ESM.docx]

Evaluation of intraocular gas using magnetic resonance imaging after pars plana vitrectomy with gas tamponade for rhegmatogenous retinal detachment

Makoto Gozawa M.D.^1^, Masayuki Kanamoto Ph.D.^2^, Shota Ishida Ph.D.^2^, Yoshihiro Takamura M.D., Ph.D.^1^, Kentaro Iwasaki M.D.^1^, Hirohiko Kimura M.D., Ph.D.^3^, Masaru Inatani M.D., Ph.D^1^

^1^ Department of Ophthalmology, Faculty of Medical Sciences, University of Fukui, 23-3 Shimoaizuki, Matsuoka, Eiheiji, Yoshida, Fukui 910-1193, Japan

^2^ Radiological Center, University of Fukui Hospital, 23-3 Shimoaizuki, Matsuoka, Eiheiji, Yoshida, Fukui 910-1193, Japan

^3^ Department of Radiology, Faculty of Medical Sciences, University of Fukui, 23-3 Shimoaizuki, Matsuoka, Eiheiji, Yoshida, Fukui 910-1193, Japan

**Supplementary file 2 Gas contact rates in each case in supine position**

|  | Supine Position | | | | |
| --- | --- | --- | --- | --- | --- |
| Case | Superior-posterior | Superior-anterior |  | Inferior-posterior | Inferior-anterior |
| 1 | 0 | 100 |  | 0 | 62.0 |
| 2 | 3.9 | 100 |  | 12.1 | 100 |
| 3 | 0 | 100 |  | 0 | 100 |
| 4 | 24.2 | 100 |  | 18.7 | 100 |
| 5 | 0 | 100 |  | 15.1 | 100 |
| 6 | 0 | 13.9 |  | 0 | 69.8 |
| 7 | 0 | 98.9 |  | 0 | 88.6 |
| 8 | 13.6 | 100 |  | 17.2 | 100 |
| Mean ± SE | 5.2 ± 3.2 | 89.1 ± 10.7 |  | 7.9 ± 3.1 | 90.1 ± 5.5 |

SE = standard error
